# Treatment of newly diagnosed B-cell origin primary CNS lymphoma with systemic R-IDARAM chemotherapy and intrathecal immunochemotherapy

**DOI:** 10.18632/oncotarget.8370

**Published:** 2016-03-25

**Authors:** Liren Qian, Chunhui Zhou, Jianliang Shen, Jian Cen, Wenjie Yin

**Affiliations:** ^1^ Department of Hematology, Navy General Hospital, Beijing, PR China; ^2^ Department of Neurosurgery, Navy General Hospital, Beijing, PR China

**Keywords:** R-IDARAM, primary CNS lymphoma, diffuse large B-cell lymphoma, rituximab, immunochemotherapy

## Abstract

**Background:**

Primary central nervous system lymphoma (PCNSL) is a rare subtype of non-Hodgkin's lymphoma (NHL). The aim was to evaluate response rate, progression free survival (PFS), overall survival (OS), and toxicity in PCNSL after systemic R-IDARAM and intrathecal immunochemotherapy with deferred radiotherapy.

**Results:**

The response rate was 94% with 17 (89%) complete responses and 1 (5%) partial responses. Follow-up time is from 5 to 63 months (median, 39 months). Median survival has not been reached. 3-year overall survival and progression-free survival rates were 84.2% (CI 72.6% to 99.8%) and 63.2% (CI 41.4% to 73.8%). Systemic toxicity was mainly hematologic. Neurocognitive and neuromotor deterioration as a result of treatment occurred in only one patient (5%).

**Patients and Methods:**

From September 2010 to June 2015, 19 consecutive patients with PCNSL (median age, 54 years) were enrolled into a pilot phase II study evaluating immunochemotherapy without radiotherapy. The patients were accrued to a chemotherapy regimen that incorporated rituximab, idarubicin, dexamethasone, cytarabine (Ara-c) and methotrexate (MTX) combined with intrathecal rituximab, MTX, dexamethasone and Ara-c.

**Conclusions:**

The results indicate that R-IDARAM regimen with intrathecal immunochemotherapy is generally well tolerated and produces a high complete response rate and survival rate.

## INTRODUCTION

Primary central nervous system lymphoma (PCNSL) is a rare subtype of non-Hodgkin's lymphoma(NHL) which accounts for 3.3% of all brain tumors [1, 2], less than 5% of all lymphomas [3, 4]. With the increase of organ transplantation and immunosuppressant application, elevated incidence of HIV/AIDS, etc, its incidence has been steadily increasing during the last three decades and the rising rate of its incidence is the first among intracranial tumors [5]. PCNSL is confined to the brain, eyes, leptomeninges or spinal cord in the absence of extracerebral tumor manifestation and metastases [1].

Diffuse large B-cell lymphoma(DLBCL) accounts for about 90% of PCNSL. The remainders include Burkitt's lymphoma, T-cell rich B-cell lymphoma, peripheral T-cell lymphoma and rarely ‘low-grade’ B-cell lymphoma [4, 6]. Because of its rarity and drugs’ inability to cross the blood-brain barrier, optimal treatment is limited. Overall survival rate in patients with PCNSL and long-term survival is much lower than the same histological type of lymphoma involving peripheral lymphoid organs. Despite these obstacles, substantial progress has been made. A growing number of clinical trials have shown the efficacy of several treatment strategies. Application of high-dose methotrexate (HD-MTX) was associated with significantly improved outcome because of its ability to cross the blood-brain barrier(BBB). However, clinical trials have not shown a clear advantage in the use of MTX doses above 3 g/m^2^. A phase II trial resulted in a 64% response rate before whole-brain radiotherapy by using 1 g/m^2^ of MTX monotherapy plus six doses of intrathecal MTX (12 mg per dose) [7]. Another phase II trial resulted in a 74% response rate by using 8 g/m^2^ of MTX monotherapy [8]. Results of the two clinical trials are comparable. But a dose of 8 g/m^2^ MTX was frequently needed for a dose reduction during the course of the treatment because of its toxicity, including renal impairment, myelotoxicity, mucositis, etc. Radiation therapy has also been used for decades for the treatment of PCNSL, but its role has been diminishing over the last years. Standard doses of radiation can lead to serious age-related neurotoxicity, such as cognition, memory and other functions impairment, brain atrophy, leukoencephalopathy, endocrine disorders, dementia, etc [9]. Eckhard Thiel et al. found that no significant difference in overall survival was recorded when whole brain radiotherapy was omitted from first-line chemotherapy in patients with newly diagnosed PCNSL [10]. The progression-free survival benefit afforded by whole brain radiotherapy has to be weighed against the increased risk of neurotoxicity in long-term survivors.

Optimum treatment for patients with PCNSL remains challenging and at present there is no universally accepted therapeutic approach for patients with newly diagnosed disease. It is clearly necessary to investigate new therapeutic methods on PCNSL.

R-IDARAM chemotherapy regimen(rituximab, idarubicin, dexamethasone, cytarabine, MTX) was applied in very few patients until now [2, 4, 11]. Here, we report a study enrolled 19 patients treated with systemic R-IDARAM immunochemotherapy combined with intrathecal rituximab, MTX, dexamethasone and Ara-c. This study addressed the question of whether this combined systemic and intrathecal immuochemotherapy results in long time survival and durable tumor responses.

## RESULTS

### Patient characteristics and treatment

The results of this paper come from an interim analysis. 19 patients were enrolled into this study. The median age was 54 years (range, 24 to 75 years), and the median Karnofsky performance score (KPS) was 70 (range, 20 to 100). Further characteristics of patients are listed in Table 1. Histologic types and location of lymphoma in each patient are listed in Table 2. During enrollment, 2 additional patients were not included because of lacking informed consent (*n* = 1), bacterial pneumonia (*n* = 1). All but 3 patients (15%) had CSF involvement at presentation. Complete treatment without any modification was given to 16 patients. Reasons for incomplete treatment were early death (1 patient), discontinuation of therapy by the patient (2 patients). MTX dose was reduced in 2 patients (up to 30% in one patient and up to 20% in the other one).

**Table 1:** Patient characteristics

	No. of patients	%
Age, years		
Median	54	
Range	24–75	
Sex		
Male	9	47%
Female	10	53%
Immunophenotype		
B-cell origin	19	100%
T-cell origin	0	0%
CSF involvement	3	15%
KPS at diagnosis		
Median	70	
Range	20–100	
80–100	4	21%
60–70	12	63%
40–50	2	11%
20–30	1	5%

Abbreviation: KPS, Karnofsky performance score.

**Table 2:** Histologic types and location of lymphoma in each patient

Patient No	Histologic type	location
1	DLBCL	Left basal ganglia
2	DLBCL	Bilateral temporal lobe, Left frontal lobe
3	DLBCL	Left lateral ventricle
4	DLBCL	Right parietal lobe
5	DLBCL	Left insula, Left frontal lobe, Left basal ganglia
6	DLBCL	Left cerebellum
7	DLBCL	Right parietal lobe
8	DLBCL	Left frontal lobe
9	DLBCL	Right internal capsule, Right pons, Right cerebral peduncle
10	DLBCL	Left cerebellum
11	DLBCL	Right temporal lobe
12	DLBCL	Bilateral lateral ventricle
13	DLBCL	Left temporal lobe
14	DLBCL	Left temporal lobe
15	DLBCL	Left lateral ventricle, Corpus callosum
16	DLBCL	Left frontal lobe, Septum pellucidum, Caudate nucleus
17	Low-grade B-cell lymphoma	Basal ganglia, Splenium of corpus callosum
18	DLBCL	Bilateral cerebellum
19	DLBCL	Right frontal lobe

Abbreviation: DLBCL, Diffuse large B-cell lymphoma.

### Treatment response, OS and PFS

17 patients (89%) achieved CR, one (5%) achieved PR, and one patient (5%) progressed under therapy (Table 3). The patient with PR was irradiated after 6 cycles chemotherapy as a result of nephrotoxicity, and received no further treatment. The patient with progressed disease was recommended to receive further chemotherapy after 2 cycles, but the patient refused to receive further treatment because of financial problem.

**Table 3:** Response to treatment

Resopnse	Whole Group (*N* = 19)	
	No.	%
Complete response	17	89%
Partial response	1	5%
Progressive disease	1	5%
Treatment-related deaths	0	0%

**Figure 1:**
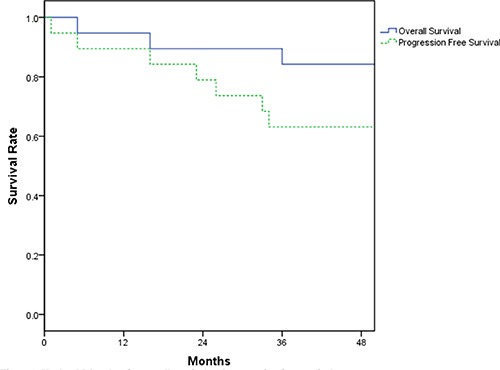
Kaplan-Meier plots for overall survival and progression-free survival

Figure 1 shows the Kaplan-Meier survival curves for overall survival and disease-free survival. The median follow-up time is 39 months (range 5 to 63). Median survival and median progression-free survival for our patient population has not yet been reached. 3-year overall survival and progression-free survival rates were 84.2% (CI 72.6% to 99.8%) and 63.2% (CI 41.4% to 73.8%), respectively. 3 patients died from disease progression (2 patients) and relapse (1 patient).

### Toxicity

Clinical and laboratory toxicities are listed in Table 4.

**Table 4:** Clinical and laboratory toxicities

Toxicity	Grade 1	Grade 2	Grade 3	Grade 4
Clinical toxicities				
Neurologic				
Neurocognitive	1			
Neuromotor		1		
Gastrointestinal				
Constipation	1			
Nausea	8			
Vomiting	4			
Mucositis	3	2		
Other				
Fatigue	7	2	1	
Laboratory toxicities				
Hematologic				
Hemoglobin		8	6	3
Leukocytes		8	8	3
Platelets	5	2	1	4
Neutrophils	5	4	5	5
Renal				
Creatinine	4			
Urea nitrogen	4			
Hepatic				
Transaminases	9	5	3	
Bilirubin	1	2	1	
Other				
Glucose	8	3	2	1

104 cycles of chemotherapy were administered (mean, 5.4 cycles per patient; range, one to six cycles of therapy). There were no treatment-related deaths. Mucositis and renal and hepatic toxicities were minimal and not therapy limiting. Twenty-three cycles of chemotherapy were complicated by grade 3 or 4 neutropenia.

Formal neurocognitive testing was performed on nine patients. One of these patients experienced neurocognitive and one experienced neuromotor deterioration after treatment. Of the other seven patients formally tested, no evidence of neurocognitive decline was noted. Of the patients not formally tested, no patient experienced severe neurocognitive toxicity.

## DISCUSSION

The IDARAM regimen was found to be effective in 7 patients with CNSL by Moreton P et al. [2]. and was modified by Mehmet Yilmaz et al. to R-IDARAM in 3 patients with PCNSL [12]. Our group further combined R-IDARAM with radiotherapy in 3 patients with PCNSL [11]. No further study was reported about the efficacy and safety of R-IDARAM on PCNSL patients with overall survival and progression free survival. In 2016, Maciocia P et al. treated diffuse large B-cell lymphoma with secondary CNSL involvement which also showed encouraging efficacy and well tolerance [13]. This study evaluated the efficacy and safety of R-IDARAM chemotherapy and intrathecal immunochemotherapy in patients with PCNSL.

We found high response rates and improved progression-free and overall survival among patients. Treatment with R-IDARAM plus intrathecal rituximab was well tolerated. Overall survival and progression-free survival rates at 3 years were 84.2% and 63.2% which are superior to results achieved with radiotherapy [14] or with high-dose MTX alone [8, 15]. Our results are also superior to the results of some polychemotherapy trials [16] and to the results of some combination chemotherapy and radiotherapy trials [9, 17, 18]. In the study by Shah GD et al. they evaluated the efficacy of combined immunochemotherapy with reduced whole-brain radiotherapy for newly diagnosed PCNSL [9]. 2-year overall and progression-free survival were 67% and 57% in the study which were lower than the results in this study. Our results are also comparable to the results of recent polychemotherapy trials [19, 20]. Antonio Omuro et al. evaluated R-MPV followed by high-dose chemotherapy with TBC and autologous stem-cell transplant for newly diagnosed PCNSL [20]. In their study, median PFS and OS were also not reached. Two-year PFS and OS were 79% and 81%, which are comparable to the results in this study.

Chemotherapy was the first line treatment for PCNSL if patients are sufficiently fit. The most important problem of drug deliver is the blood-brain barrier. MTX, Ara-c, idarubicinol (metabolite of idarubicin), dexamethasone have been demonstrated can penetrate blood-brain barrier at certain concentrations [21–23]. To achieve therapeutic concentration of MTX in the brain, high doses are required (i.e. at least 1.5 g/m^2^). We modified the regimens by reducing methotrexate to 2 g/m^2^ previously [11] to reduce its toxicity without reducing its efficacy. In this study, we further modified the regimens by adding intrathecal rituximab which may increase the efficacy without increasing its toxicity.

Rituximab is an anti-CD20 monoclonal antibody. The addition of Rituximab to CHOP has improved the survival of patients with DLBCL. However, as a large protein it has poor penetration into the CNS as measured by CSF levels. Rituximab has been detected in the CSF at concentration ranging from 0.1% to 4.4% of serum levels after intravenous administration in patients with CNS lymphoma [24]. Rituximab transport to the CSF may occur via leaking across areas of blood-brain barrier breakdown in the lymphoma and/or macromolecular vesicular transport of the antibody across an intact blood-brain barrier [25]. Rituximab may improve the survival of patients with PCNSL [9], but the precise role of rituximab in PCNSL remains controversial and unclear [26]. Intrathecal rituximab combined with systemic reduced dose chemotherapy has been demonstrated in other studies reducing the toxicity of systemic chemotherapy but increased the efficacy [27, 28].

In this study, we added rituximab intravenously and intrathecally which showed good efficacy and safety. No related toxicity was shown in this study by intrathecal rituximab, which may be an optimized method for us to make rituximab penetrate the blood-brain- barrier.

In conclusion, immunochemotherapy is a promising treatment approach for patients with newly diagnosed PCNSL, and our findings suggest that R-IDARAM combined with intrathecal immunochemotherapy increases CR rates. We also found intrathecal rituximab combined with systemic reduced dose chemotherapy may reduce the toxicity of systemic chemotherapy and increase the efficacy. But further studies with large patient sample are needed to verify whether the addition of rituximab to standard chemotherapy results in a significant improvement in patient outcome. This study supports future clinical trials of R-IDARAM plus intrathecal immunochemotherapy for PCNSL.

## PATIENTS AND METHODS

### Eligibility criteria

All eligible patients had newly diagnosed histologically proven non-Hodgkin's lymphoma (NHL) according to the Revised European-American Lymphoma and WHO classification [29]. Patients with lymphoma that involved sites other than the brain, meninges, CSF, or the eyes were not included. Exclusion criteria were age less than 18 years or greater than 75 years, inadequate bone marrow capacity (defined as neutrophils < 1.5 × 10^9/L, platelets < 100 × 10^9/L, and hemoglobin level < 8 g/dL), known cause of immunosuppression (ie, HIV type I infection), any previous malignancy, creatinine clearance below 60 mL/min, heart insufficiency (New York Heart Association classification of heart disease class IIIB or IV), uncontrolled infection, or noncompensated active pulmonary or liver disease. Patients previously treated for PCNSL, except by corticosteroids, were not included. All patients provided informed consent. The study was approved by the ethics committees of Navy General Hospital and was registered in ClinicalTrials.gov: NCT02657785.

### Baseline studies

All patients entered into the study were evaluated the following examinations: complete history and physical examination, blood count, electrolyte levels, tests of renal and liver function that included a 24-hour creatinine clearance, HIV, EBV and hepatitis B serologies, lactate dehydrogenase level, and serum protein electrophoresis. Staging consisted of magnetic resonance imaging (MRI) of the brain, lumbar puncture, bone marrow biopsy and cytology, chest and abdominal computed tomography, and ophthalmologic evaluation including plitlamp examination.

### Treatment protocol and study design

Between September 2010 to June 2015, 19 consecutive patients were enrolled into a single-center pilot phase II study (Navy General Hospital, China). Treatment consisted of six chemotherapy cycles separated by intervals of 3 weeks between each cycle. Details of the protocol are listed in Table 5. The R-IDARAM chemotherapy regimen was applied to all patients. This regimen incorporated rituximab 375 mg/m^2^ (day 1), idarubicin 10 mg/m^2^ (day 2 and 3); dexamethasone 100 mg/m^2^ (12 h. infusion in day 2, 3 and 4); Ara-c 1 g/m^2^ (1 h. infusion in day 2 and 3); MTX 2 g/m^2^ (6 h. infusion in day 4 with folinic acid rescue), Intrathecal rituximab 10 mg, MTX 15 mg, dexamethasone 5 mg and Ara-c 50 mg once a week. Colony stimulating factor (150 ug/m^2^) was also started 48 h after systemic chemotherapy. Chemotherapy cycles were given at 3-weekly intervals.

**Table 5:** Chemotherapy protocol for primary CNS lymphoma

Chemotherapy	Day 1	Day 2	Day 3	Day 4	Day 5	Day 6	Day 7	Day 8
Rituximab	+							
Idarubicin		+	+					
Dexamethasone^*^		+	+	+				
Cytarabine		+	+					
Methotrexate^&^				+				
Intraventricular Chemotherapy^#^	+							+

* Dexamethasone was infused for 12 h.

& Methotrexate was infused for 6 h.

# Intraventricular Immunochemotherapy regimen includes rituximab 10 mg, MTX 15 mg, dexamethasone 5 mg and Ara-c 50 mg once a week.

Colonystimulating factor (150 ug/m^2^) was also started 48 h after systemic chemotherapy.

### Evaluation of response and toxicity

The primary efficacy parameters were response rate, complete response rate, partial response rate, survival, and progression-free survival. Response was determined after every two chemotherapy cycles by contrast-enhanced MRI of the brain. Neuroradiographic response criteria as defined by Macdonald et al. [30]. Survival and progression-free survival were measured from the study accrual date, which was generally within a few days of first drug administration. Toxicity was graded using the National Cancer Institute Common Toxicity Criteria.

### Statistics

The results of this paper come from an interim analysis. The primary end points were 3-year progression-free survival (PFS) and acute treatment-related toxicity. Secondary end points included response rate, Overall survival, PFS, long-term neurocognitive outcome and treatment morbidity. The sample size was calculated on the basis of primary end point of progression-free survival, and the number of patients required was based on the assumption of an exponential distribution of events. Response rate for efficiency was monitored by using the design of Thall et al. [31]. The probability that the true complete response proportion is at least 0.60 was calculated, and if this was less than 0.10, then entry of patients would cease. The incidence of unacceptable toxicity, defined as grade 4 nonhematologic toxicity, was also monitored. If, at any point, the interim results gave a 95% probability that the true proportion of unacceptable toxicity was greater than 0.2, then entry of patients would cease. The Kaplan-Meier method was used to summarize the time-to-event variables.
